# Dynamic neutrophil lipidome remodeling during induction of NETosis

**DOI:** 10.1126/sciadv.aec9891

**Published:** 2026-06-24

**Authors:** Patrick Münzer, Cristina Coman, Gundula D. Lingens, Nina N. Troppmair, Jesse A. Michael, Reuben S. E. Young, Stefanie Rubenzucker, Jens Martin, Jann Arden, Melina Fischer, Nils Sülzle, Ferdinand Kollotzek, Monika Zdanyte, Claudia Hornef, Shane R. Ellis, Oliver Borst, Robert Ahrends

**Affiliations:** ^1^ DFG Heisenberg Group Cardiovascular Thrombo-Inflammation and Translational Thrombocardiology, Tübingen, Germany.; ^2^ Department of Cardiology and Angiology, University of Tübingen, Tübingen, Germany.; ^3^ Institute of Analytical Chemistry, University of Vienna, Vienna, Austria.; ^4^ Molecular Horizons and School of Science, University of Wollongong, Wollongong, NSW, Australia.; ^5^ DFG Cluster of Excellence 2180 ‘Image-guided and Functional Instructed Tumor Therapy’ (iFIT), University of Tübingen, Tübingen, Germany.

## Abstract

Neutrophil extracellular trap formation (NETosis) affects a wide variety of clinically relevant human diseases. Although lipid remodeling is essential for neutrophil function and membrane rupture during NETosis, the neutrophil lipidome and its dynamics have not been characterized. Thus, we establish a quantitative lipidome of human neutrophils comprising 1048 species across nine orders of magnitude and map its remodeling during NETosis. NET formation caused profound alterations in the phosphatidylinositol, phosphatidic acid, diacylglycerol (DG), and lyso-glycerophospholipid levels. Calcium- and reactive oxygen species–dependent NETosis pathways displayed distinct lipidomic trajectories yet converged on the significance of phospholipid lipase networks. Pharmacological inhibition of this networks altered lipid composition and markedly impaired NETosis, while DG treatment revoked the effect. Together, our findings reveal lipid remodeling as a fundamental determinant of NETosis and identify interconnected and dependent phospholipid lipase networks with downstream DG-dependent signaling as a potential therapeutic target in NET-associated diseases.

## Teaser

Lipid remodeling by phospholipid lipase networks is a fundamental determinant of NETosis.

## INTRODUCTION

Polymorphonuclear neutrophils (PMNs) represent the most abundant subset of immune cells and mediate important antimicrobial activities. Among other mechanisms, neutrophils can expel decondensed chromatin structures designated neutrophil extracellular traps (NETs). First described in 2004 as a pivotal part of the innate immunity (*1*), the formation of NETs (NETosis) has since been linked to the development and progression of a wide variety of clinically relevant human disorders. Beyond their involvement in generating autoantibodies in rheumatoid arthritis and heparin-induced thrombocytopenia (*2*, *3*), NETs are highly implicated in arterial and venous thrombosis (*4*, *5*), myocardial ischemia/reperfusion injury (*6*), infective endocarditis (*7*), and myocarditis (*8*). Moreover, under pathophysiological conditions, NET formation can be triggered by diabetes (*9*) and cancer (*10*), thus contributing to cancer progression and metastasis (*11*). Despite the remarkable clinical significance, the complex cellular mechanisms underlying NETosis remain ill-defined.

NET formation is a strictly regulated sequence of cellular events leading up to the disassembly of nucleosomes by peptidylarginine deiminase 4 (PAD4)–dependent (*12*, *13*) and myeloperoxidase (MPO)–dependent (*14*) mechanisms. Ultimately, entropic chromatin swelling culminates in the rupture of the nuclear envelope and the plasma membrane (*15*) with subsequent expulsion of neutrophilic chromatin together with prothrombotic and proinflammatory factors (*16*). Although more physiological stimuli such as bacteria, activated platelets, and fungi exist, most effective inducers of these processes in vitro are the calcium ionophore ionomycin and phorbol 12-myristate 13-acetate (PMA), which activate PAD4 or trigger the production of reactive oxygen species (ROS) with subsequent MPO activation, respectively (*17*). Considering the rupture of the neutrophilic plasma membrane may occur hours after NET induction and an observed gradual increase in plasma membrane permeability (*18*, *19*), a highly regulated dynamic shift in the lipid composition of the plasma membrane likely contributes significantly to NETosis. However, a detailed description of the quantitative lipidome and dynamic lipid profile in neutrophils especially upon NET induction is still missing.

Besides a study on the importance of cholesterol for neutrophil function (*20*), research on the exact distribution of specific phospholipid classes remains insufficient. Whereas some studies identified phosphatidylcholine (PC) as the most abundant class in neutrophils and phosphatidylserine (PS) and phosphatidylethanolamine (PE) at similar levels (*21*), others did not analyze PS levels at all and report PE as the most prominent lipid class in neutrophils (*22*). Despite these discrepancies, there is a broad consensus that the lipidome of neutrophils is distinct and characterized by an abundance of vinyl ether and ether lipids, believed to be oxidized and halogenated (*23*), thus providing protection against oxidative stress, including ferroptosis. Moreover, it seems that neutrophils contain a diverse array of glycosphingolipids, such as gangliosides and members of the (neo)lacto series, which appear to be absent in other immune cells (*24*), and may contribute to pathogen recognition and interactions with the humoral immune system. Thereby, especially, the glycosphingolipid lactosylceramide seems to be highly prevalent in lysosomal granules of neutrophils (*25*), possibly affecting the clearance of pathogens in line with the innate immune response.

To date, most of the studies on neutrophil lipids have merely examined broad categories, often omitting entire classes or capturing only a fraction of the lipidome. Detailed and quantitative analyses of the molecular lipid species comprising the neutrophil lipidome and highlighting the plasma membrane composition are still lacking, and none of the recent studies investigated dynamic lipid changes in neutrophils upon induction of NETosis. Thus, this study aimed to characterize in a quantitative lipidomics approach the lipid profile of neutrophils and the dynamic changes during NETosis. Consequently, we were able to quantitatively define the most prominent lipid classes in neutrophils and illustrate dynamic lipid changes and pertinent lipid-driven signaling pathways essentially mediating the cellular process of NETosis.

## RESULTS

### Characterization of the quantitative neutrophil lipidome

**Fig. 1.**
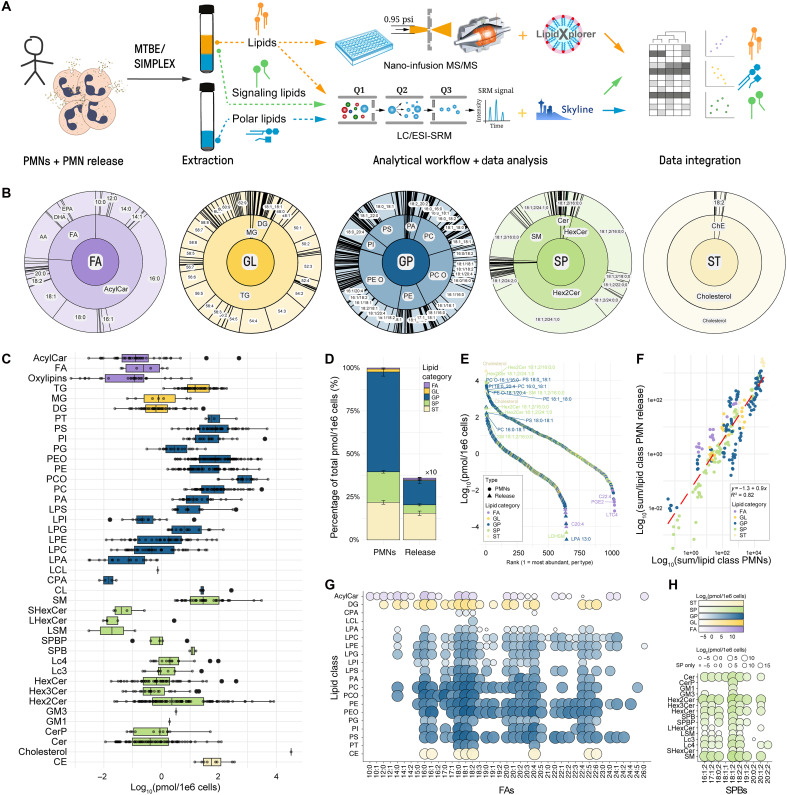
Quantitative lipidomics reveals a complex and multifaceted lipidome for PMNs covering a wide, dynamic range spanning nine orders of magnitude. (**A**) Quantitative lipidomics platform covering membrane lipids, signaling lipids, and lipids for energy demands. (**B**) Quantitative distribution of the five lipid categories analyzed in mol % per category; inner circle illustrates class distribution, and the outer circle shows the distribution of species within each class. (**C**) Boxplots presenting the dynamic range of the PMN lipidome at the resting stage, where each entry represents a lipid species. Quantities of five lipid categories [fatty acids (FAs), sphingolipids (SPs), glycerolipids (GLs), glycerophospholipids (GPs), and sterols (STs)] spanning 41 lipid classes and 1048 lipid species over a concentration range of nine orders of magnitude are shown. Each bar is composed of all quantified lipid species within the respective class. SM, sphingomyelin; SPBP, sphingoid base-phosphate; SPB, sphingoid base; HexCer, hexosylceramide; Hex2Cer, dihexosylceramide; Cer, ceramide; GM3, monosialodihexosylganglioside; GM1, monosialotetrahexosylganglioside; Lc3, lactotriaosylceramide; Lc4, neolactotetraosylceramide/paragloboside; TG, triacylglycerol; DG, diacylglycerol; PS, phosphatidylserine; PI, phosphatidylinositol; PEO, phosphatidylethanolamine-ether; PE, phosphatidylethanolamine; PCO, phosphatidylcholine-ether; PC, phosphatidylcholine; PG, phosphatidylglycerol; PA, phosphatidic acid; LPA, lyso-PA; LPI, lyso-PI; LPG, lyso-PG; LPE, lyso-PE; LPC, lyso-PC; CL, cardiolipin; CE, cholesterol-ester. (**D**) Comparison of the quantitative composition of the PMN cell and its lipid release at the resting state in mol %; colors represent categories. (**E**) S-plot of the cellular and PMN release lipid concentrations displaying more and less abundant molecules across nine orders of magnitude. (**F**) Linear regression demonstrating similar patterns at lipid class level between PMNs and its release. (**G**) Fatty acyl distribution across different classes including GL, GP, ST, and acyl-carnitines. (**H**) Long-chain base (LCB) distribution for the analyzed SP classes. All data represent mean values of six biological replicates.

To quantitatively profile the lipidome of naïve circulating primary neutrophils, we used a multiplatform lipidomics workflow (Fig. 1A). In unstimulated neutrophils, the most abundant lipid categories, in descending order, were phospholipids [glycerophospholipid (GP)], sterols (STs), fatty acids (FAs), glycerolipids (GLs), and sphingolipids (SPs) (Fig. 1B). Within GPs, PC, PE, and PS predominated, whereas phosphatidylinositol (PI), phosphatidylglycerol (PG), and phosphatidic acid (PA) were comparatively minor (Fig. 1, B and C). More than 50% of the entire class of PE and PC is covered by ether lipids.

Neutrophils exhibited a lipidomic dynamic range spanning nine orders of magnitude (Fig. 1, C and E), with cholesterol (32743 pmol per million cells) as the most and prostaglandin E_2_ (PGE_2_) as one of the least (0.0021 pmol per million cells) abundant molecules in PMNs (Fig. 1E), whereas cholesterol (2538 pmol per million cells) and lyso-sphingomyelin (LSM; 0.0022 pmol per million cells) present as the highest and among the lowest levels within the PMN release (Fig. 1E). Compared with megakaryocytes, neutrophils displayed a less complex lipid repertoire yet were more complex than platelets, where 30 species account for ~90% of the lipidome (fig. S1) (*26*, *27*). The distribution among classes and species is comparable between the PMN membrane lipidome and secreted lipids (Fig. 1, D to F, and fig. S2A), with the exception of a lower lipid amount (~4% of the PMN lipidome), less species and increased levels of cholesterol and hexosylceramides detected in the PMN release. Upon lipid release, the lipid composition of vesicular membranes diverged from that of plasma membranes, indicating a selective remodeling of the neutrophil lipidome during vesiculation. Notably, neutrophil complex lipids consisted primarily of the 10 most abundant FAs (Fig. 1G), including 16:0, 18:0, 18:1, 18:2, and 20:4, and the *sn* positional and double-bond isomer analysis of the most abundant PCs and PEs displays species-specific distributions (figs. S3 and S4).

The levels of bound docosahexaenoic acid (DHA) and eicosapentaenoic acid (EPA) were noticeably lower in neutrophils compared with those in naïve B cells, megakaryocytes, or platelets (*27*), indicating a limited capacity for producing lipid mediators derived from these precursors (fig. S2, B and C). SP analysis revealed a high diversity of long-chain bases (LCBs; sphingoid bases), including the major LCBs 18:1, 18:2, 17:1, 16:1, and 18:0 (Fig. 1H), while the bound FAs ranged from 14:0;0 to 26:1;0 (fig. S2, D and E).

Together, these results establish a comprehensive and detailed reference lipidome for circulating naïve neutrophils. They illustrate an unprecedented lipid architecture, characterized by ether lipid enrichment, limited DHA/EPA availability, and distinct remodeling upon lipid release, features that may significantly shape the distinct inflammatory and signaling function of neutrophils. The novelty and magnitude of these findings will surely pique the interest of the scientific community.

### Characterization of the lipidome upon NET induction with ionomycin

NETs can be induced in vitro by coculture with bacteria and activated platelets, which, however, both comprise an own and distinct lipidome (*27*). Thus, to investigate the dynamic lipidome only in neutrophils upon NET induction, primarily isolated neutrophils were stimulated for 1, 30, and 120 min with well-established NET inducers ionomycin or PMA.

**Fig. 2.**
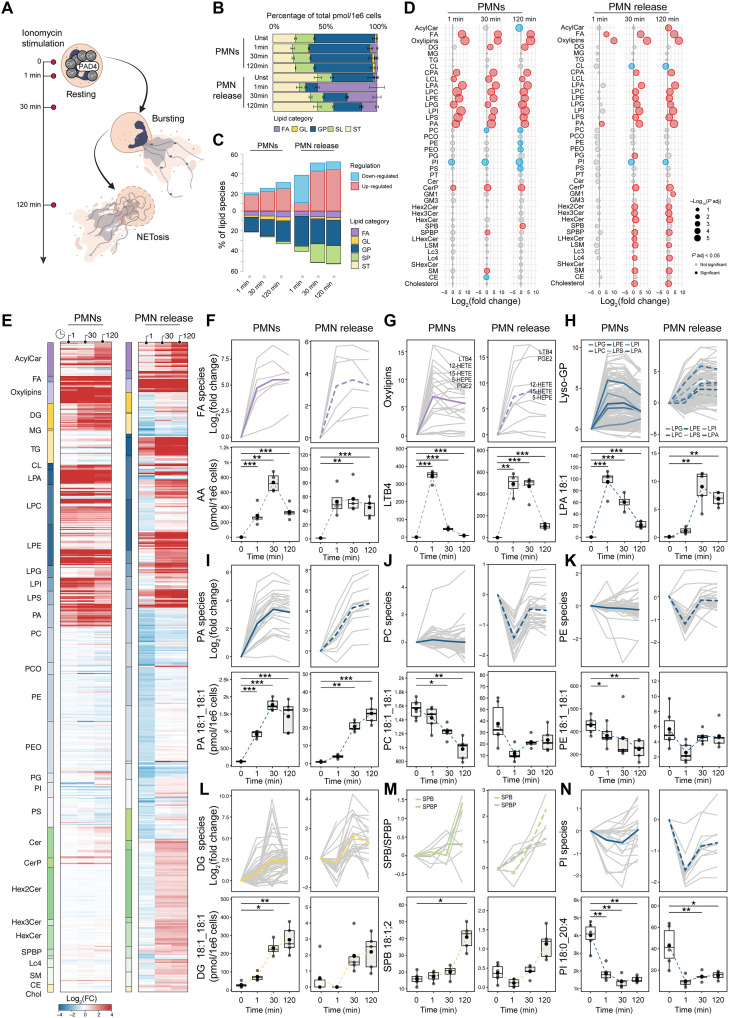
A rapid dynamic lipidome remodeling of PMNs is triggered by Ca^2+^ influx during NET formation. (**A**) Timeline of sampling after NET induction using 4 μM ionomycin triggering Ca^2+^ influx. (**B**) Category-based lipidome remodeling of PMNs and release after NET induction showing glycerophopholipids, GL SPs, STs, and fatty acyls. (**C**) Quantitative assessment of the number and direction of species regulated in each class during NETosis at 1, 30, and 120 min. (**D**) Ratio plots presenting class dynamics at 1, 30, and 120 min (left to right) for the cellular and the released lipidome. The ratios are depicted as log_2_ fold changes. Here, the most prominent modulations of the lipidome at class level during NET formation and release are displayed. Significant changes are colored in red for up- and blue for down-regulated classes. (**E**) Heatmap at lipid species level during NETosis with class distinction and color-coded category assignments. Only lipid species which were significantly changing (*P*_adj_ < 0.05) in at least one of the comparisons are displayed. FC, fold change. (**F** to **N**) Class-wise lipid regulation upon ionomycin treatment with examples of molecular lipid species for each class. (F and G) Fewer complex lipids such as fatty acyls, oxylipins, and lyso-GPs. (I to K) Membrane lipids, and major phospholipid classes (such as PA, PCs, and PEs). (L to N) DGs, LCBs, and PIs as potential signaling lipids. Examples have been provided for each panel. Mean fold changes for individual lipid species are shown as gray lines, the class average is depicted in color. The time points were always compared to the unstimulated quantities [paired *t* test, *P*_adj_ < 0.05 (*n* = 3 to 5 matched biological replicates per condition)].

During ionomycin treatment (Fig. 2A), the calcium ionophore increases intracellular Ca^2+^ levels via transportation across plasma and organelle membranes. In neutrophils, this leads to the activation of the calcium-dependent effector PAD4 with subsequent citrullination of histones and lastly DNA decondensation. In general, increasing intracellular Ca^2+^ levels affect downstream calcineurin–nuclear factor of activated T cell signaling and, most likely, phospholipase A_2_ (PLA_2_) activation. However, apart from fatty acyls and lyso-phospholipids, most lipid categories in PMNs are not displaying noticeable shifts within 2 hours of activation (Fig. 2B). The most abundant classes, including PC and PE, as well as less abundant classes, such as PS and PG, are not regulated within the first 30 min (Fig. 2D). The exception lies in the down-regulation of PI by 50%, leading to increased levels of lyso-PI (LPI) both inside and outside the cell, likely through PLA_2_ activity (*28*). This activity is underscored by higher intra- and extracellular levels of lyso-PC (LPC), lyso-PS (LPS), lyso-PE (LPE), lyso-PG (LPG), and lyso-PA (LPA) (Fig. 2H), whereas lyso-sphigomyelin levels remain unchanged in PMNs (Fig. 2D, right). PLA_2_ activity drives an overall increased production of free arachidonic acid (AA), DHA, docosapentaenoic acid (DPA), and EPA (Fig. 2, E and F, and table S1) as the most abundant polyunsaturated fatty acids (PUFAs) in neutrophils, correlating with a rapid up-regulation of oxylipins within the first minute (Fig. 2G) (*29*). The comparatively minute impact of PLA_2_ on highly abundant PUFAs is likely related to the presence of ethers instead of esters in two-thirds of all PLs, reducing the accessibility toward PLA_2_. Notably, all PLA_2_-dependent changes occur within the first minute of activation (Fig. 2, F to H). However, as a large contributor to AA release, the precursor PI 18:0_20:4 was identified similarly as in platelet activation (Fig. 2N) (*27*).

The most significant change in complex phospholipids in PMNs is the up-regulation of phosphatic acid (PA) peaking at 30 min (Fig. 2D), likely connected to phospholipase D (PLD) activity. As indicated by the decreasing levels of specific species, PC more than PE (Fig. 2, J and K) could be used for PA generation. It appears that PA regulation coincides with a diacylglycerol (DG) increase (at 2 hours), which is induced by DG kinase (DGK) and phospholipase β/γ (PLCβ/γ). The DG increase is essential for neutrophil chemotaxis (*30*) and a key element in the production of complex GP and GL, an effect detectable across almost all species (Fig. 2L). Exclusive of the delayed esterase activity, oxidation of PUFAs via the 5/15-lipoxygenase (5/15-Lox) or cyclooxygenase-1/2 (Cox-1/2) pathways becomes evident early on, leading to increased levels of hydroxyeicosatetraenoic acids (HETEs) and PGE_2_ (Fig. 2, E and G). Similar trends can be observed overall upon NET induction when comparing the cellular to the released lipidome (Fig. 2, F to N); however, SP levels are substantially amplified (Fig. 2, C and D), indicating endoplasmic reticulum–derived vesicular release. While major SP classes [such as sphingomyelin (SM) and ceramides] remain unchanged (Fig. 2D, left), sphingosine is increased within the cell, and S1P (SPBP) levels are elevated in the PMN release, indicating SP signaling (Fig. 2, D and M) upon PMN activation via ionomycin, which is likely used to attract more neutrophils to the site of activation, fostering their adhesion and further degranulation (Fig. 2, C and M) (*31*, *32*). The magnitude of change is more prominent in the PMN release (Fig. 2B) with increased percentages (in mol) and numbers of regulated species (Fig. 2C), reflecting the intracellular developments. Within PMNs, ionomycin induces rapid and widespread fatty-acyl remodeling, consistent with activation of calcium-dependent phospholipases. This regulation is lipid-class specific and distributed across multiple acyl compositions rather than confined to a narrow subset. Notable class-specific differences are evident: LPA shows a strong increase across nearly all species, whereas LPC and LPE display little PUFA regulation at the fatty-acyl level, and LPI exhibits only modest PUFA remodeling compared with LPA and PA (fig. S5, A and B). These findings highlight the complex and dynamic nature of neutrophil lipid metabolism during NET induction via ionomycin and demonstrate their link to cellular function and inflammatory responses, underscoring the significance and potential impact of this research.

### Characterization of the lipidome upon NET induction with PMA

**Fig. 3.**
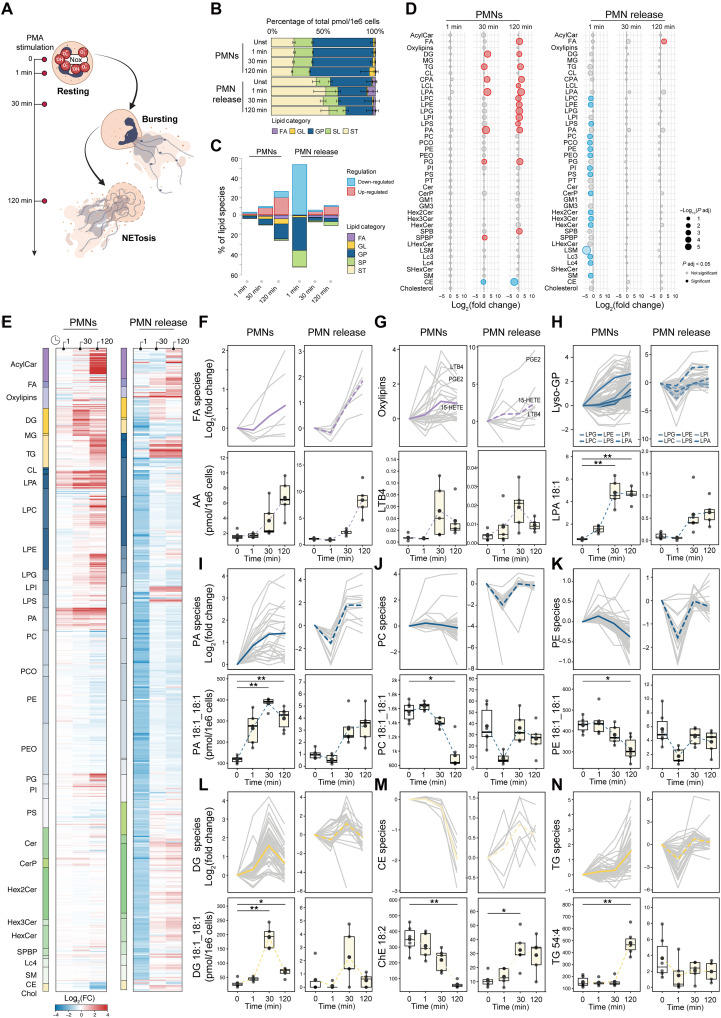
Dynamic gradual lipidome rearrangement of PMNs during NETosis triggered by DGs. (**A**) Timeline of sampling after NET induction using 100 nM PMA. (**B**) Category-based lipidome remodeling of PMNs and release after NET induction. (**C**) Quantitative assessment of the number and direction of species regulated by class during NETosis. (**D**) Ratio plots displaying class dynamics at 1, 30, and 120 min (left to right) for the cellular and the released lipidome. The ratios are displayed as log_2_ fold changes and depict considerable modulations of the PMNs and released lipidome at class level during NET formation. Significant shifts are shown in color: blue for down-regulated and red for up-regulated classes. (**E**) Heatmap at lipid species level during NETosis at 1, 30, and 120 min. Only lipid species which were significantly changing (*P*_adj_ < 0.05) in at least one of the comparisons are displayed. (**F** to **H**) Less complex lipids such as fatty acyls, oxylipins, and lyso-GPs. (**I** to **K**) Membrane lipids, and major phospholipid classes such as PA, PCs, and PEs. (**L** to **N**) DGs, ST esters, and triacylglycerols with the last two representing lipid droplet components. Examples are given for each of the panels. Mean fold changes for individual lipid species are presented as gray lines, and the average per class is depicted in color. The time points were always compared to the unstimulated quantities [paired *t* test, *P*_adj_ < 0.05 (*n* = 3 to 5 matched biological replicates per condition)].

Compared to ionomycin, PMA stimulation (Fig. 3A) triggers less regulation (Fig. 3, B to D) but also results in lower and delayed PLA_2_ activity in PMNs and the PMN release (Fig. 3D). Almost no vesicular release can be observed after PMA stimulation. In PMNs, multiple lysolipid classes are elevated after 120 min with a simultaneous increase of fatty acyls (Fig. 3, D and E). At the category level, only minor changes can be observed in PMNs (Fig. 3B), and major lipid classes like PC and PE remain overall unchanged. Lipid classes involved in GL synthesis, such as PG, PA, and DG, increase after 30 min (Fig. 3, D to F, I, and L), suggesting a similar core regulation in the lipidome as observed during ionomycin treatment. This remodeling is likely also linked to PLD activity, as suggested by the preferential decrease in specific PC species compared to PE (Fig. 3, J and K), supporting PC as a primary substrate for PA generation. With PMA, the increased DG levels are used for the production of triacylglycerols (TGs), and, consequently, lipid droplet formation takes place between 30 and 120 min upon NET induction (Fig. 3, D, E, and N). The reduction of cholesteryl esters further indicates a comprehensive remodeling of storage lipids within PMNs (Fig. 3, D, E, and M). Compared to intracellular lipid levels, the lipid release upon PMA activation displays changes only within the first min, during which many lipid classes decrease. However, after 60 and 120 min, all released lipids are stabilized with no significant changes detectable at class level (Fig. 3, C and D). Likely, the initial changes occur through stress-induced pinocytosis (*33*), commonly used for scavenging metabolites to meet any kind of energy demands. We also find fewer lipid shifts and one-fourth as many regulated lipids within the PMN releasate compared with ionomycin stimulation (Fig. 3C). PMA treatment also triggered a more gradual FA remodeling response, with modest early effects followed by pronounced regulation of PA, PI, and selective increases in LPA at later time points. Compared to ionomycin, PI levels remained relatively stable, suggesting that PMA primarily drives delayed membrane restructuring and lipid accumulation rather than acute lysophospholipid generation. A key exception is cholesteryl esters, which are consistently down-regulated across all fatty-acyl species after 120 min of PMA treatment (fig. S5, A and B).

In addition, if we review NETosis from a structural lipid perspective, neither *sn*- nor double-bond positions are altered indicating no further stereochemical rearrangements during ionomycin or PMA treatment (fig. S6).

### NET induction is characterized by a time-dependent interplay of lipases

**Fig. 4.**
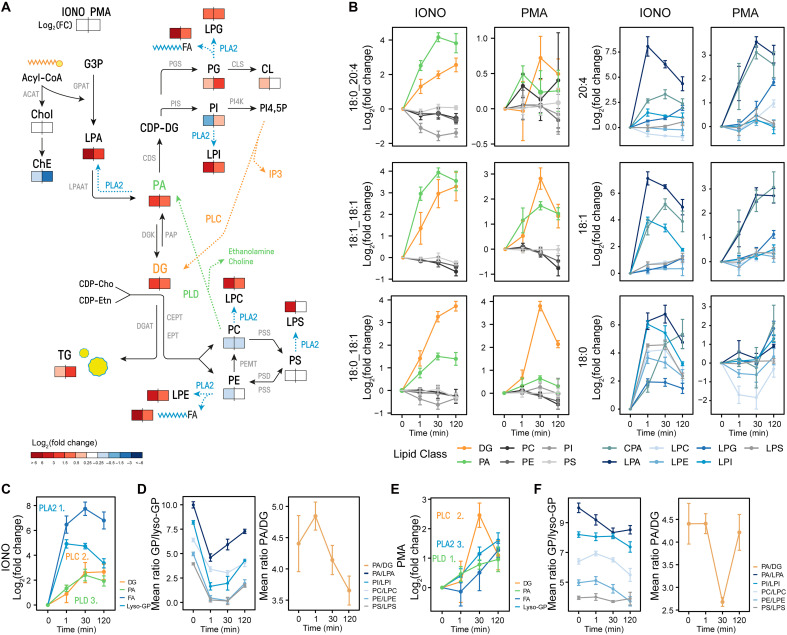
PMN lipidome remodeling is characterized by interconnected phospholipase activity. (**A**) Monitored lipid metabolism and lipase activity during PMN activation using ionomycin (IONO; left) and PMA (right); significant changes are color coded by log_2_ mean fold change scale compared to the control at 0 min; red indicates up-regulated and blue down-regulated lipid classes. (**B**) Interconnected lipid species during NETosis. Different species of DGs (orange), PA (green), and lyso-phospholipids (blue) are selected and compared to other phospholipid classes (gray). For PA and DG, mono-, di-, and polyunsaturated FAs, and, for the lyso-phospholipids, saturated, mono-unsaturated, and polyunsaturated FAs are displayed. (**C**) Summary of lipid class profiles reflecting PLA_2_ (blue), PLC (yellow), and PLD (green) activity during ionomycin treatment; class-level summaries of lyso-GP, FA (PLA_2_), DG (PLC), and PA (PLD) were used for readout. (**D**) The ratio of GPs to lyso-GPs is displayed indicating early PLA_2_ activity during ionomycin treatment, PA/LPA (dark blue), PI/LPI (blue), PC/LPC (light blue), PE/LPE (light blue), and PS/LPS (gray); PA-to-DG ratio is reflecting onset of PLC versus PLD activity during PMN activation. (**E**) Summary of lipid class profiles reflecting PLA_2_, PLC, and PLD activity during PMA treatment; class-level summaries of lyso-GP, FA (PLA), DG (PLC), and PA (PLD) were used for readout. PA-to-DG ratio reflects shifts in phospholipase activity between PLD and PLC during PMN activation. (**F**) The ratio of GPs to lyso-GPs is displayed indicating delayed PLA_2_ activity during PMA activation, PA/LPA (dark blue), PI/LPI (blue), PC/LPC (light blue), PE/LPE (light blue), and PS/LPS (gray); PA-to-DG ratio is reflecting onset of PLC versus PLD activity during PMN activation (*n* = 5 biological replicates).

The lipidome shift during PMN activation is characterized by a combination of PLA_2_, PLC, and PLD activity (Fig. 4A and figs. S7 and S8), which, in the presence of ionomycin and PMA, triggers oxidative processes within the 5/15-Lox or Cox-1/2 pathways as indicated by the increased levels of oxylipins (Figs. 2D and 3D) such as LTB4 or PGE_2_ (Figs. 2G and 3G). The activity of these lipases ultimately leads to elevated levels of PA and DG (Fig. 4B), resulting in positive reinforcement through the protein kinase C (PKC) pathway.

When enzyme activity is plotted using the relevant precursor-to-product ratios, the transient nature of lipase activity becomes evident (Fig. 4, D and F). During ionomycin activation PLA_2_ activity rises quickly and reaches peak activity after 1 min of stimulation (Fig. 4B), which is indicated by the steep increase of lyso-GPs and FAs (Fig. 4, B and C). PLD and PLC activity increases between 30 and 120 min, demonstrated by increasing levels of PA and DG (Fig. 4, B and C). As PLC levels continue to rise, PLD gradually declines toward the 120-min mark.

In contrast, during PMA activation, PLD increases earliest, followed by PLC, and then PLA_2_ (Fig. 4, F and E), which reaches its maximum activity at 120 min, while PLC activity decreases (Fig. 4, B and E). Overall, both stimuli demonstrate at 120 min an overall elevated PLA_2_, PLD, and PLC activity, which is likely essential for the proper induction of NETosis.

### Effect of phospholipase signaling on in vitro NETosis and lipid dynamics

**Fig. 5.**
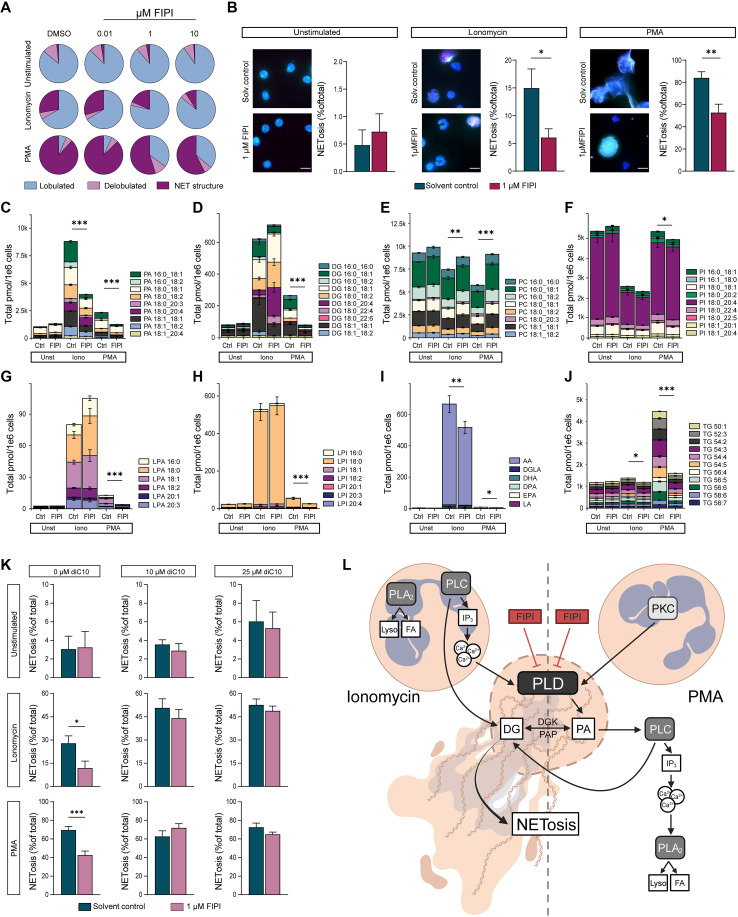
PLD inhibition reveals quantitative and qualitative traits of PA and DG signaling in NETosis. (**A**) Percentage of unstimulated, ionomycin-stimulated (4 μM), or PMA-stimulated (100 nM) PMNs at various cellular stages of NETosis (*n* = 6) in the absence [dimethyl sulfoxide (DMSO)] or presence of FIPI at the indicated concentration. (**B**) Percentage of NETs (*n* = 6) in unstimulated, ionomycin-stimulated (4 μM), or PMA-stimulated (100 nM) PMNs in the absence (solvent control) or presence of 1 μM FIPI. (**C** to **J**) dissected lipid class distribution visualized at species level during PLD inhibition and NETosis triggered by PMA and ionomycin stimulation. The most abundant lipid species of the following lipid classes are depicted: (C) PA, (D) DGs, (E) PCs, (F) PIs, (G) LPA, (H) LPI, (I) FAs, and (J) TGs. The comparison of the lipid classes is provided by corresponding controls. The data display the average of the biological replicates, paired *t* test, *P*_adj_ < 0.05 (*n* = 6 to 7 matched biological replicates per condition). (**K**) Percentage of NETs (*n* = 6) in unstimulated, ionomycin-stimulated (4 μM), or PMA-stimulated (100 nM) PMNs in the absence (solvent control) or presence of 1 μM FIPI and concurrent treatment with diC10 at the indicated concentrations (0, 10, and 25 μM), *P* < 0.05. (**L**) Summary of the interconnected phospholipase network modulating the PMN lipidome and NETosis.

Subsequently, we addressed the physiological function of phospholipase signaling on NET formation, of which PLD specifically has been identified as a crucial element in lipid modifications. We investigated the effect of pharmacological PLD inhibition via specific inhibitor 5-fluoro-2-indolyl deschlorohalopemide (FIPI) on NETosis in circulating human neutrophils by using in vitro assays. The pretreatment of PMNs with FIPI evoked noticeable changes in cellular hallmarks of NET formation such as nuclear lobulation and plasma membrane rupture. In the absence of FIPI, a clear increase in NET structures with a simultaneous decrease in nuclear lobulation was observed after ionomycin or PMA stimulation (Fig. 5A). In a concentration-dependent manner, pretreatment with FIPI resulted in an increased number of lobulated neutrophils and markedly decreased NET structures in activated PMNs, when compared to the solvent control (dimethyl sulfoxide) (Fig. 5A), which is altogether indicative of reduced NETosis. Correspondingly, pretreatment with 1 μM FIPI resulted in a significantly impaired ionomycin- and PMA-induced NETosis compared to the solvent control, whereas FIPI treatment had no effect on unstimulated naïve PMNs (Fig. 5B).

One of the identified hallmarks of lipid associated dynamics upon NET induction is the PLD-dependent generation of PA and DG (Fig. 4B). Thus, in a next set of experiments we investigated the effect of pharmacological PLD inhibition on the lipidome of PMNs. When compared to the solvent control, the presence of 1 μM FIPI leads to reduced PA levels upon ionomycin or PMA stimulation (Fig. 5C). Thereby, mainly di-unsaturated, mono-unsaturated, and saturated PA species (e.g., PA 16:0_18:1) were affected by PLD inhibition (Fig. 5C), suggesting a corresponding substrate specificity. Notably, while total DG levels were significantly down-regulated after PMA stimulation in the presence of FIPI, they remained unaffected upon ionomycin stimulation (Fig. 5D). However, even with overall consistent DG levels, remodeling occurs in response to PLD inhibition after ionomycin treatment when compared to the solvent control (Fig. 5D). Again, comparable with the affected PA species, predominantly mono-unsaturated and saturated DG species emerging from mono-unsaturated and saturated PA species were down-regulated upon FIPI treatment and ionomycin-induced NETosis (Fig. 5D). In addition, DG lipid species containing the same PUFA released by PLA_2_ are increased (Fig. 5D), which indicates DGK activity via PLC shunt compensating for the loss of DG containing saturated and mono-unsaturated such as DG 18:1_18:1, DG 16:0_16:0, and 16:0_18:1. Adding the PLD inhibitor validates our point that PA is PC derived, as shown by the restoration of PC levels after FIPI treatment (Fig. 5E). PLA_2_ activity also seems to be influenced by PLD inhibition as reflected by the reduced LPA, LPI, and FA levels during PMA-induced NET activation (Fig. 5, G to I), which points toward an interconnected downstream phospholipase network. In contrast, PLD inhibition during ionomycin-induced NET activation affects less PLA_2_ activity, as evidenced by the fact that PI levels are not restored (Fig. 5F), indicating that PLA_2_ function is maintained under these conditions. In addition, an effect on TG synthesis and consequently lipid droplet production was observed in the PMA-stimulated PMNs after PLD inhibition with TG levels not changing upon PLD inhibition (Fig. 5J).

PA and particularly mono-unsaturated and saturated DG species are well-established upstream effectors of PKC, which plays a pivotal role in ionomycin- as well as PMA-induced NETosis (*34*). Moreover, because FIPI treatment in ionomycin- and PMA-induced NETosis resulted in markedly decreased mono-unsaturated and saturated DG species, we investigated the effect of *sn*-1,2-didecanoylglycerol (diC_10_) treatment on in vitro NET formation in PLD-inhibited neutrophils. As depicted in Fig. 5K, the treatment with diC_10_ concurrent with PLD inhibition completely restored NETosis after ionomycin or PMA stimulation (Fig. 5K), demonstrating the importance of PA- and DG-induced signaling in the process of NET formation (Fig. 5I), which ultimately underscores our suggested model of action (Fig. 5L).

## DISCUSSION

NETs not only are linked to infectious diseases but also affect the development and progression of thrombo-inflammatory pathologies such as thrombosis (*4*, *35*, *36*) or ischemic and inflammatory heart diseases (*6*–*8*) and are additionally connected to cancer (*10*) and diabetes (*9*), all of which contribute significantly to morbidity and mortality worldwide (*37*). In this context, besides the commonly used inductors ionomycin and PMA, NETs can also be triggered by bacteria, activated platelets, and fungi for instance. Consequently, NETosis is highly relevant at the pathophysiological level, and potential clinical applications for prophylaxis as well as treatment of NET-related disorders remain underdeveloped. Unfortunately, the exact cellular and molecular signaling mechanisms underlying NETosis are insufficiently defined. In particular, the quantitative lipidome and the role of dynamic lipid alterations during NETosis are often neglected, although the lipidome of neutrophils seems to be distinct (*22*) and a broad range of neutrophil functions such as phagocytosis, adhesion, secretion of microbicidal compounds, and NETosis are crucially dependent on the properties and lipid composition of the plasma membrane. Therefore, in the underlying study, we used state-of-the-art lipidomics workflows to characterize the quantitative lipidome of human neutrophils and its dynamic alteration during ionomycin- or PMA-induced NETosis. This distinct and comprehensive approach uncovered a lipidomic dynamic range spanning nine orders of magnitude in naïve neutrophils with ether-linked lipid species representing the predominant GP classes (Fig. 1), which is in accordance with previous reports (*22*) and corroborates our methodological approach. In addition, the lipidome comprises a highly dynamic range (nine orders) and an impressive variety of components (1048 species, across 41 classes), indicating a large functional diversity among lipids. The large proportion of hexosyl and di-hexosylceramides and the relatively low proportion of cholesterol and SM indicate a rather flexible membrane. However, although our analysis captures the most abundant glycosphingolipid classes, the substantial structural diversity and complexity of PMN glycosphingolipids limit the comprehensiveness of their coverage in the present study. In addition, we characterized the dynamic changes in the lipid landscape during NETosis. Distinctive, stimulus-dependent patterns of membrane remodeling were observed after ionomycin and PMA activation; however, the main lipid classes modified including PA, DG, lyso-GP, and fatty acyls proved similar. In this context, our study identified phospholipase networks and PLD specificity toward saturated, di-unsaturated, and mono-unsaturated fatty acyls as crucial elements of in vitro NETosis upon stimulation with ionomycin or PMA and altogether established a comprehensive and detailed reference lipidome for circulating naïve neutrophils. Not to mention, although the stimulation of neutrophils by bacteria, activated platelets, and fungi all culminate in the activation of PAD4 (*12*) or the generation of ROS, these more physiological stimuli possibly evoke different lipidome dynamics during NETosis. In particular, because different bacteria cause different and distinct intracellular signaling pathways in neutrophils (*38*), these possible alternative lipidome dynamics have to be investigated in more detailed further studies.

PLD is strongly expressed in immune cells including neutrophils (*39*), and its activation is implicated in phagocytosis and oxidant production (*40*). PLD predominantly generates the anionic phospholipid PA, which is known to alter the lipid composition of the plasma membrane’s inner leaflet causing a negative membrane curvature. Although ionomycin- and PMA-induced NETosis manifest as distinct lipidomes and lipid dynamics, both inducers converged on the generation of PA and DG (Fig. 4A). In other immune cells, modified membrane curvatures caused by PA are known to directly impact cellular shape and exocytotic and phagocytotic processes (*41*). Membrane curvature was demonstrated to serve as key regulator of the spatial distribution of the ubiquitously expressed mechanosensitive ion cannel Piezo1 in living cells (*42*), which was just recently identified as a positive regulator of shear-induced NETosis (*43*). Thus, at least, under shear stress, PA-induced changes in plasma membrane curvature may directly contribute to the increased plasma membrane permeability and rupture observed at distinct stages of NETosis (*18*). Nevertheless, further studies are required to determine the exact role of PA and mechanotransduction during NETosis, considering the neuronal-specific Piezo2 channel seems to be negatively affected by endogenous PA (*44*). Intriguingly, in the past years, the involvement of NETs in cancer and diabetes has been well acknowledged (*9*, *10*), including reports on the major role of PLD in metastasis and chemoresistance (*45*) and metabolic disorders such as diabetes (*39*, *46*), underscoring the importance a PLD-dependent signaling mechanism underlying NETosis.

Besides affecting physical plasma membrane properties, PA also serves as an important signaling lipid in the plasma membrane (*47*), and its protein binding properties are affected by cholesterol (*48*), an ST known to affect NETosis (*20*) and that is the most abundant molecule in naïve neutrophils (Fig. 1C, bottom). The importance of PA protein binding properties is further corroborated by the observation that PA binds directly to actin-related protein 3, thus affecting actin cytoskeleton dynamics and subsequently leukocyte adhesion (*49*), whereas, especially, the early and quick rearrangement of the actin cytoskeleton represents a defined major hallmark of NETosis (*18*).

Both PA and DG reside in an equilibrium via phosphatidate phosphatases (PAP) and DGK driving PKC activation. Moreover, PLD activation seems to affect the release of ROS from neutrophils upon PMA stimulation (*50*), a mechanism most likely involving PKC-dependent signaling (*51*, *52*). Thereby, in an isoform-dependent manner, PKC signaling is undoubtedly linked to the formation of NETs upon calcium ionophore and PMA stimulation (*17*, *34*, *53*). The involvement of PLD-dependent signaling in the regulation of PKC activity and its localization in immune cells is increasingly evident, because the simultaneous generation of PA and DG is essential for the translocation of a specific PKC isoform to the plasma membrane (*54*). Our results demonstrated that the pharmacological inhibition of PLD by FIPI impaired ionomycin- and PMA-induced NETosis in vitro in a concentration-dependent manner (Fig. 5, A and B). This effect was completely reversed in the presence of the DG analog and PKC activator diC_10_ (Fig. 5K), which is in accordance with data showing a restored oxidase activation in neutrophils upon PLD inhibition (*40*). These findings most likely point to the involvement of generated PA and DG in PKC signaling during ionomycin- and PMA-induced NETosis. However, further investigations are required to unravel the exact role of PA- and DG-induced NETosis, because diC_10_ could be transformed to PA via a DGK-dependent mechanism.

Conclusively, this study delineates comprehensively the quantitative lipidome of naïve neutrophils and the dynamic lipid alterations underlying NETosis upon iono- and PMA stimulation. Furthermore, PLD-dependent PA and DG generation was linked to NETosis. Thus, the presented results unravel mechanistic insights into the molecular signaling of NETosis, which will contribute to our understanding of the multifaceted nature of NET formation and its connection to clinically relevant human disorders. From a translational approach, our results demonstrate that pharmacological PLD inhibition or lipid-associated manipulation of NETosis may be a promising strategy for the prophylaxis and treatment of NET-associated inflammatory diseases.

## MATERIALS AND METHODS

### Materials and standards

Acetonitrile (ACN), methanol (MeOH), formic acid, and water were purchased in liquid chromatography–mass spectrometry (LC-MS) grade from Biosolve (Valkenswaard, The Netherlands). Ammonium acetate (NH4Ac), ammonium formate (AF), chloroform (CHCl_3_), phosphoric acid, potassium chloride, sodium chloride, SDS, dithiotreitol, trifluoroacetic acid, iodoacetamide, triethylammonium bicarbonate, and *tert*-butyl methyl ether (MTBE) were obtained from Sigma-Aldrich (Steinheim, Germany), and acetic acid (HAc) was obtained from Carl Roth (Karlsruhe, Germany). Isopropanol (IPA) was purchased from Merck (Darmstadt, Germany), and tris(hydroxymethyl)-aminomethane from Roche Diagnostics (Mannheim, Germany). All lipid standards were purchased from Cayman Chemical (Ann Arbor, USA) or Avanti Polar Lipids (Alabaster, USA), and labeled carnitine standard set B was purchased from Euriso-top (Saarbrücken, Germany).

### Ethical issues

All blood donors gave informed consent. The study was approved by the institutional ethics committee (032/2024BO2) and complies with the declaration of Helsinki and good clinical practice guidelines.

### Neutrophil isolation

Human neutrophils were isolated using gradient centrifugation as previously described (*55*). Concisely, EDTA-anticoagulated blood was layered on Histopaque-1119 and spun for 21 min at 1100*g* without deceleration. After a washing step, the resulting cell pellet was resuspended in Hanks’ balanced salt solution (HBSS) and layered on an 85/80/75/70/65% Percoll gradient column before being centrifuged for 21 min at 1100*g* without deceleration. The resulting neutrophil layers were washed again with HBSS and subsequently resuspended in phenol-red free RPMI medium containing 10 mM Hepes. Neutrophil concentration and purity were determined using a hemocytometer (Sysmex KX-21N).

### Preparation of neutrophils for quantitative lipidomics

The desired cell density (2 × 10^4^ to 3 × 10^4^/μl) was adjusted using phenol-red free RPMI medium containing 10 mM Hepes. Cells (2 × 10^6^ to 3 × 10^6^) were stimulated in the absence or presence of PLD inhibitor (FIPI, 1 μM) at the indicated time points. After stimulation, samples were spun down for 10 min at 400*g*, and the pellet and the supernatant were immediately frozen in liquid nitrogen and stored at −80°C until further processing.

### In vitro NET assay

In vitro NET assays were performed as described previously (*55*). Concisely, isolated human neutrophils were adjusted to a cell density of 3 × 10^2^/μl using phenol-red free RPMI medium containing 10 mM Hepes and 0.05% bovine serum albumin. Cells (3 × 10^5^) were added on coverslips to a 24-well plate with the DG analogon (diC_10_) in the absence or presence of PLD inhibitor (FIPI) for 30 min at 37°C and 5% CO_2_. Afterward, cells were stimulated for 4 hours at 37°C and 5% CO_2_ with ionomycin (4 μM) or PMA (100 nM) and subsequently fixed with 1.3% paraformaldehyde for 30 min at room temperature. After washing with PBS, permeabilization and blocking, samples were stained overnight with primary antibodies against citrullinated histone H3 (no. ab219407, 1:1000) and neutrophil elastase (no. GTX72042, 1:1000). Following two washing steps, the corresponding secondary antibodies (nos. A31574 and A21428, 1:1500) were incubated for 2 hours at room temperature, and, after two additional rounds of washing, the samples were lastly mounted using ProLong Diamond Antifade with 4′,6-diamidino-2-phenylindole (no. P36962). For analysis of NET formation, at least three microscopic images were taken from each sample, and the percentage of NET formation was calculated using ImageJ.

### Lipid extraction

Neutrophil pellet and supernatant were used for lipid extraction following the SIMPLEX protocol previously described by Coman *et al.* (*56*). In short, 225 μl of MeOH and the internal standard mixture (Mouse Splash, CerMix II, chol-d7, carnitine set B, and an in-house mixture containing odd-chain LPI, LPG, LPA, LPS, AA-d11, DHA-d5, EPA-d5, 13-HODE-d4, 12-HETE-d8, 15-HETE-d8, 9-HODE-d4, LTB4-d4, PGD2-d4, PGE_2_-d4, GB3-d7 18:1;2/18:0;0, GM3-d7 18:1;2/18:0;0, and GM1a-d7 18:1;2/18:0;0) were added to the samples and subjected to ultrasonication for 10 s on ice water. Next, 750 μl of MTBE was added, and samples were incubated for 1 hour at 950 rpm at 4°C. To induce phase separation, 188 μl of water was added. After a 10-min centrifugation step at 10,000*g* at 4°C, the upper organic phase (containing GPs, GLs, SPs, and STs) was carefully removed and dried under a gentle nitrogen flow. To complete protein precipitation, 527 μl of MeOH was added to the lower aqueous phase, and samples were stored for 2 hours at −20°C, following centrifugation for 30 min at 12,000*g* at 4°C. The lower aqueous phase (containing FAs and acidic SLs) was collected and dried under a gentle nitrogen flow.

### Lipid measurements and analysis

#### Shotgun lipidomics

Lipid extracts were resuspended in IPA/MeOH/CHCl_3_ (4:2:1, v/v/v) with 7.5 mM NH4Ac and then infused via the TriVersa NanoMate ion source (Advion Biosciences) into an Exploris 240 (Thermo Fisher Scientific) mass spectrometer. The following settings were used for positive and negative modes: ionization voltage, +1.25/−1.25 kV; backpressure, 0.95 psi; ion transfer capillary temperature, 250°C; S-lens level of 60%; and EASY-IC was enabled. In both modes, full mass spectrometry (MS) spectra were acquired with a resolution of 240,000 followed by a data-independent acquisition (DIA) for precursor masses at an interval of 1.001 Da. Tandem MS (MS/MS) spectra were acquired with a resolution of 60,000, and the precursor isolation window was 1 Da.

#### LC-HRMS analysis for GPLs and GLs of PMN release

Lipid extract (5 μl) resuspended in butanol (BuOH):IPA:H_2_O 8:23:69 (v/v/v) and 25 nM CUDA was loaded onto a YMCAccura Triart C18 column (150 mm by 2.1 mm, 1.9-μm particle size; YMC Europe, Dinslaken, Germany). Mobile phase A was ACN/H_2_O (60:40, v/v), mobile phase B was IPA/ACN (90/10, v/v), and both contained 10 mM AF and 0.1% formic acid. The temperatures of the autosampler and the column oven were set to 8° and 60°C, respectively, and separation was carried out with a flow rate of 0.26 ml/min with the following 30-min-long gradient: initial, 30% B; 0.0 to 3.0 min, hold 30% B; 3.0 to 15.0 min, ramp to 75% B; 15.0 to 17.0 min, ramp to 100% B; 17.0 to 30.0 min, to 5% B; and 30.1 to 35.0 min, to 30% B. The injector needle was automatically washed with 30% B.

The liquid chromatography (LC) was coupled to an Exploris 240, and data were acquired both in positive and negative ion mode. The following electrospray ionization (ESI) source parameters were applied: spray voltage, 3.4 or 2.4 kV, respectively; capillary temperature, 275°C; sheath gas flow rate, 45; auxiliary gas flow rate, 20; auxiliary gas heater temperature, 350°C; and S-lens radio frequency (rf) level, 60. Full MS spectra from 400 to 1230 mass/charge ratio (*m*/*z*) and from 400 to 1600 *m*/*z* were acquired in positive and negative modes, respectively with a resolution of 120,000, an automatic gain control (AGC) target of 1 × 10^6^, and a maximum injection time (IT) of 105 ms in one run. DDA with a top 10 experiment was used on the pool samples and acquired in separate runs for positive and negative modes. For MS/MS, a resolution of 15,000, an AGC target of 1 × 10^5^, a maximum IT of 105 ms, and a normalized collision energy (nCE) of 24 and 28 for positive and negative modes were applied.

#### Targeted lipidomics for SLs and STs

Each sample was resuspended in BuOH:IPA:H_2_O 8:23:69 (v/v/v) and 25 nM CUDA, and 7 μl was injected into the LC system. Analysis of SP and ST was performed as previously described by Peng *et al.* (*57*) and Troppmair *et al.* (*58*). Inclusion lists for targeted measurements were generated using LipidCreator (v1.2.0). Briefly, a Vanquish Flex UHPLC system (Thermo Fisher Scientific, Germering, Germany) was equipped with an Ascentis Express C18 main column (150 mm by 2.1 mm, 2.7 μm, Supelco) and fitted with a guard cartridge (50 mm by 2.1 mm, 2.7 μm, Supelco) in a column oven with a temperature of 60°C. Eluent A was ACN/H_2_O (6:4, v/v; 10 mM AF, 0.1% FA, and 5 μM PA), and eluent B was IPA/ACN (9:1, v/v; 10 mM AF, 0.1% FA, and 5 μM PA). The separation was carried out at a flow rate of 0.5 ml/min with the following 25-min-long gradient: initial (30% B), 0.0 to 2.0 min (hold 30% B), 2.0 to 3.0 min (30 to 56.1% B), 3.0 to 4.0 min (56.1 to 58.3% B), 4.0 to 5.5 min (58.3 to 60.2% B), 5.5 to 7.0 min (60.2 to 60.6% B), 7.0 to 8.5 min (60.6 to 62.3% B), 8.5 to 10.0 min (62.3 to 64.0% B), 10.0 to 11.5 min (64.0 to 64.5% B), 11.5 to 13.0 min (64.5 to 66.2% B), 13.0 to 14.5 min (66.2 to 66.9% B), 14.5 to 15.0 min (66.9 to 100.0% B), 15.0 to 19.0 min (hold 100% B), 19.0 min (5% B), 19.0 to 22.0 min (hold 5% B), 22.0 min (30% B), and 22.0 to 25.0 min (hold 30% B). The LC system was coupled to a QTRAP 6500+ (Applied Biosystems, Darmstadt, Germany). The measurements were performed in positive mode with the following ESI source settings: curtain gas, 30 arbitrary units; temperature, 250°C; ion source gas I, 40 arbitrary units; ion source gas II, 65 arbitrary units; collision gas, medium; ion spray voltage, +5500 V; declustering potential, +100 V; entrance potential, +10 V; and exit potential, +13 V. For the scheduled selected reaction monitoring (SRM), Q1 and Q3 were set to unit resolution. The scheduled SRM detection window was set to 2 min, and the cycle time was set to 0.5 s. Data were acquired with Analyst (version 1.7.2; AB Sciex), and Skyline (*59*) was used to visualize results and manually integrate signals.

#### LC-HRMS analysis for complex glycosphingolipids

Complex glycosphingolipids were analyzed using a Vanquish Horizon UHPLC system (Thermo Fisher Scientific, Germering, Germany) coupled to an Exploris 240 mass spectrometer (Thermo Fisher Scientific, Bremen, Germany). Equal volumes of organic and aqueous phases were combined, dried under nitrogen flow, and resuspended in AcN:MeOH 3:1 (v/v). Chromatographic separation was performed by injecting 7 μl of each sample onto an ACQUITY Premier Glycan BEH Amide column (150 mm by 2.1 mm, 1.7 μm; Waters, Wexford, Ireland) with an integrated VanGuard FIT guard column. The autosampler temperature was maintained at 10°C, while chromatographic separation was performed with the column oven set to 40°C. Mobile phase A was 95% 15 mM NH4Ac (pH 6.1) containing 5% IPA, whereas mobile phase B was composed of 100% ACN. Separation was achieved using a 22-min gradient at a constant flow rate of 0.2 ml/min. The gradient started at 80% B, decreased to 55% B at 6.5 min, and was maintained until 8.5 min, followed by a decrease to 40% B at 11.5 min and a hold until 13.5 min, before returning to 80% B from 13.6 to 22.0 min. Before each injection, the needle was automatically rinsed using a mixture of ACN/MeOH/H_2_O (1:1:1, v/v/v). The ion transfer capillary temperature was set to 300°C, and the vaporizer temperature was set to 340°C. Spray voltages were adjusted to +3.5 kV in positive ion mode and −2.5 kV in negative ion mode. The S-lens rf level was set to 70%. To reduce unintended precursor fragmentation, the “Mild Trapping” option was enabled. For experimental samples, full-scan MS spectra were acquired in the *m*/*z* range of *m*/*z* 1000 to 1400 in positive mode. In negative mode, spectra were recorded across an *m*/*z* range of *m*/*z* 1000 to 1400 during the first 4 min and *m*/*z* 600 to 1000 thereafter, tailored to the expected lipid class *m*/*z* range. The resolution was set to 60,000, with an AGC target of 10^6^ and a maximum IT of 105 ms.

MS/MS experiments were performed to confirm identification and for structural elucidation on pooled samples in individual acquisitions for positive and negative ionization modes using a parallel reaction monitoring (PRM) approach. This method consisted of a full MS scan spanning *m*/*z* 770 to 1370 at a resolution of 45,000, an AGC target of 10^6^, and a maximum IT of 105 ms, followed by targeted data-dependent MS/MS scans acquired at a resolution of 180,000, an AGC target of 10^5^, and a maximum IT of 400 ms. The isolation window was set to 1 *m*/*z*, and nCE was optimized using available internal standards.

#### *Targeted lipidomics for FAs*, *CerP*, *lyso-GPs, and MGs*

The analysis was performed as previously described by Rubenzucker *et al.* (*60*). The samples were resuspended in BuOH:IPA:H_2_O 8:23:69 (v/v/v) and 25 nM CUDA, and 7 μl was injected onto a YMCAccura Triart C18 column (150 mm by 2.1 mm, 1.9-μm particle size; YMC Europe, Dinslaken, Germany) fitted with a Vanquish MP35N passive preheater. The column compartment and autosampler temperatures were 45° and 8°C, respectively. Eluent A was H_2_O/ACN 80/20 (v/v) and eluent B consisted of IPA/ACN/H_2_O 60/35/5 (v/v), both containing 0.5 mM NH4Ac and 0.2% HAc. The separation was carried out at 0.4 ml/min with the following 20-min gradient: 0 to 1 min (hold 30% B), 1 to 11 min (30 to 100% B), 11 to 16 min (hold at 100% B), and 16.1 to 20 min (return to 30% B and hold for reequilibration). The measurements were performed in both positive and negative modes with the following ESI source settings: curtain gas, 35 arbitrary units; temperature, 350°C; ion source gas I, 45 arbitrary units; ion source gas II, 60 arbitrary units; collision gas, medium; and ion spray voltage, +5500 and −4500 V, respectively. For the scheduled SRM, Q1 and Q3 were set to unit resolution. The cycle time was set to 1 s, the settling time was set to 20 ms, and the minimum dwell time was set to 10 ms. Data were acquired with Analyst (version 1.7.2; AB Sciex), and Skyline (*59*) was used to visualize results and manually integrate signals.

### Lipid identification and quantification

All spectra were imported by LipidXplorer (1.2.8) into a MasterScan database under the following settings: mass tolerance, 5 parts per million (ppm); *m*/*z* range, 400 to 1050 and 400 to 1200 for positive and negative modes, respectively; minimum occupation, 0.15; and intensity threshold, 5 × 10^3^. Lipid identification was carried on as described by Herzog *et al.* (*61*) by matching the *m*/*z* of the monoisotopic peaks to the corresponding elemental composition constrains. The Molecular Fragmentation Query Language queries were compiled to match precursors and fragment ions to confidently recognize lipid species. LipidXplorer output was processed by lxPostman [Lipidomics Informatics for Life Sciences (LIFS)] and in-house developed R scripts (RStudio 23.09.1+494). All identified lipids were normalized to their corresponding class-specific or structurally related deuterated internal standards. TGs and cardiolipins were structurally identified based on MS/MS fragmentation but quantified using precursor ion intensities relative to the respective class-specific internal standard (reported at the lipid species level rather than molecular species level). Subsequently, lipid quantities were normalized to cell count.

SPs (Cer, HexCer, Hex2Cer, Sulfo-HexCer, LSM, LCBP, LCB, and SM), STs (ST and SE), oxylipins, FAs, endocannabinoids, MGs, and selected lyso-GPs were identified and quantified by LC-MS analysis. Peak integration from targeted measurements was performed using Skyline (v21.1.0.146) (*59*). Lipid species abundance was calculated using peak areas and quantified according to the respective internal standard (Ceramide/Sphingoid Internal Standard Mixture II; cholesterol-d7) or as described by Rubenzucker *et al.* (*60*).

The LC–high-resolution mass spectrometry (HRMS) data analysis of the PMN release was based on the identification list obtained from the shotgun measurements of the PMNs. The lipid species–specific fragments were generated by LipidCreator and monitored in Skyline for peak integration. The MS2-based identification was performed on the pool samples of PMNs, PMN release, and plasma that were acquired in the DDA mode, and the lipid species were lastly quantified on the basis of the MS1 level. The peak areas were normalized to the ones of the corresponding class-specific standards.

Glycosphingolipid LC-MS data were visualized and manually integrated using Skyline (version 25.1.0.142). Isotopic correction was performed using a custom R script (RStudio version 2024.12.0 + 467; RStudio Team), and automated postacquisition processing was conducted using Konstanz Information Miner (KNIME; version 5.2.5) (*62*). Lipid species were annotated on the basis of retention time and class-specific headgroup fragment ions. As internal standards specific for Lc3 and Lc4 were not available, definitive discrimination between glycan isomers was not possible. However, previous studies (*63*) describing glycosphingolipid profiles in PMNs have reported the presence of lacto/neolacto-series species but not asialo series isomers. Therefore, the detected species were provisionally assigned to the lacto/neolacto series on the basis of their chromatographic behavior and MS/MS fragmentation characteristics. Quantification was performed by class-specific standards or relative to GB3-d7 for Lc3 and Lc4. All lipid quantities were normalized to the cell number in a KNIME (*62*) or R workflow.

### Isomer-resolved MS analysis

Dried lipid extracts were shipped on dry ice from Vienna, Austria, to Wollongong, Australia, in 200-μl glass inserts housed in amber-glass vials. Upon arrival, samples were stored at −80°C until analysis. On the day of measurement, extracts were equilibrated to room temperature and reconstituted in 20 μl of 4:1 IPA:MeOH. Aliquots were transferred into a 96-well plate (TwinTec, Eppendorf) and mixed with 2.5 mM sodium acetate in MeOH at a 1:1 (v/v) ratio to prepare for nanoelectrospray ionization. Ionization was performed using a TriVersa NanoMate (Advion) operated at 1.4-kV spray voltage and 0.4-psi gas pressure. The ion source was coupled to a modified Orbitrap Fusion Tribrid mass spectrometer (Thermo Fisher Scientific) that allowed passive introduction of ozone into both the high-pressure dual-ion trap and the ion routing multipole. Ozone was generated using a Titan-30UHC high-concentration ozone generator (Absolute Ozone) via dielectric barrier discharge of high-purity oxygen, yielding inline ozone concentrations of 220 g/Nm^3^.

Data were acquired using a DIA method adapted from Michael *et al.* (*64*). Positive ion mode was used with the following scan events: (i) full MS1; (ii) MS^3^ collision-induced dissociation/ozone-induced dissociation (CID/OzID) triggered by neutral loss of the PC headgroup ([M + Na − 183.0661]+); (iii) MS^3^ CID/OzID triggered by neutral loss of the PE headgroup ([M + Na − 141.0191]+); and (iv) MS^2^ OzID. CID/OzID events were stepped in 2-Da intervals across *m*/*z* 574 to 1036 with a 1-Da isolation window. At the MS^2^ level, precursor ions were subjected to a normalized collision energy (NCE) of 45 V for 5 ms, and the resulting headgroup-loss product ion was reisolated for MS^3^ analysis (dual-ion trap ozonolysis, NCE = 0 V, 400 ms). MS^2^ OzID scans were stepped through 2-Da intervals across *m*/*z* 650 to 950 with a 1-Da isolation window, followed by ion routing multipole ozonolysis for 2.5 s at NCE = 1 V.

### Data analysis of lipid double bonds and processing thresholding

For *sn*-isomer species, data were analyzed as recently described. (*64*). For double-bond isomers, data were extracted from the Thermo .raw files using Xcalibur v 3.0.63 for selected lipid species and then processed manually using Excel. Data are presented as the percentage contribution of double-bond–specific fragment intensity compared to fragments from all isomers. Given the highly complex nature of the lipid extracts, isobars were expected to be prevalent within the mass spectrum, so precursor ions were specified on the basis of theoretical *m*/*z* values, and, then, characteristic OzID neutral loss fragments were calculated using a 5-ppm threshold for assignment. The aldehyde and Criegee ions needed to reach a combined signal-to-noise ratio of 3:1 to be identified, and the assignment needed to be present in two of three sample replicates -to be included in the graphical visualizations. In addition, SEM with a 95% confidence interval was calculated per assignment, and, if error exceeded the calculated mean value, the assignment was discounted.

For analysis of *sn*-isomers, MS3 scans in the .raw files were processed through the ALEX123 pipeline from Michael *et al.* (*64*). The list of validated *sn*-isomers is generated algorithmically from all combined treatment and control files considering appropriate CID/OzID fragments detected at sufficient frequency and accurate mass (±2.5 ppm following file-dependent alignment). The “canonomer” label refers to phospholipid isomer where the longer (or if identical length, more unsaturated) of two acyl chains is in the *sn*-2 position.

### Visualization and statistical analysis

For all statistical analyses and plotting of the data, we used in-house scripts generated with R v4.3.1 (https://R-project.org). Datasets were assessed for normal distribution using the Shapiro-Wilk test (*n* = 5 to 6). For lipidomics analysis, all statistical comparisons were performed using paired *t* tests on log_2_-transformed lipid abundance values to account for the matched experimental design, in which identical donors were analyzed across experimental conditions. Log_2_ transformation was applied before statistical testing to stabilize variance and improve distributional properties of the data.

Paired comparisons were conducted between each treatment condition, and the corresponding unstimulated control within matched biological replicates and mainly log_2_ (fold changes) are displayed. For a limited number of lipid species, statistical comparisons were based on three to four paired biological replicates due to the condition-dependent absence of detectable signal for some lipid species (1.9% with *n* = 3, 11.8% with *n* = 4 of total comparisons); all other comparisons were analyzed using five to seven paired replicates as described in the figure legends. For visualization purposes, when absolute lipid abundances (picomol per 1 × 10^6^ cells) are presented in the figures, statistical analyses were performed exclusively on log_2_-transformed data.

To control for multiple testing, *P* values were adjusted using the Benjamini-Hochberg false discovery rate procedure across the 1048 tested lipid species. For the microscopy analysis shown in Fig. 5, a Welch’s *t* test was performed.

### Network analysis

Relationships between lipid species across stimulation conditions were investigated using a correlation-based network similarity approach. Quantitative lipid abundance data obtained from unstimulated and stimulated cells at all investigated time points were used for this analysis. Similarity between lipid species was determined by calculating Pearson correlation coefficients across all measured conditions for each possible lipid pair, using pairwise complete observations to account for missing values. Lipid pairs exhibiting strong similarity in their abundance profiles (correlation coefficient ≥ 0.95) were considered functionally associated and used to define network connections. Within the resulting network, each lipid species was represented as a node, while edges weighted according to the correlation coefficient indicated highly correlated temporal and treatment-dependent abundance patterns. To facilitate biological interpretation, additional lipid descriptors, including lipid category and class annotations as well as fold-change values relative to unstimulated samples (log_2_ transformed), were incorporated as node attributes but were excluded from correlation calculations. Network construction and visualization were performed using Cytoscape (version 3.9.1) (*65*).
